# Patients’ experiences with shared decision-making in home-based palliative care – navigation through major life decisions

**DOI:** 10.1186/s12904-024-01434-2

**Published:** 2024-04-17

**Authors:** Sandra Jahr Svendsen, Ellen Karine Grov, Katrine Staats

**Affiliations:** 1 Lillestrøm Municipality, Lillestrøm, Norway; 2 Faculty of Health Sciences, Department of Nursing and Health Promotion, Oslo Metropolitan University, Kjeller, Norway; 3 Faculty of Health Sciences, Department of Nursing and Health Promotion, Oslo Metropolitan University, Pilestredet, Norway

**Keywords:** Palliative care, Decision-making, Nursing, Home care services

## Abstract

**Background:**

This study addresses the issue of shared decision-making (SDM) in a Norwegian home-based palliative care setting. The significance of patient involvement in SDM is widely acknowledged, and many patients want to participate in decisions about care and treatment. Yet, it remains a need for more knowledge regarding the initiators and approaches of SDM in the context of home-based palliative care, particularly from the patients’ perspective. The aim of this study is to understand patients' experiences and preferences for SDM in home-based palliative care, seeking to enhance the quality of care and direct the planning of healthcare services.

**Methods:**

We used a qualitative explorative design. A hermeneutic approach was employed, and data was collected through in-dept interviews with 13 patients.

**Results:**

The study uncovered an overarching theme of "Navigating to reach own decisions," comprising three sub-themes: “To be trapped in life without decisions to act on”; “To surrender to others and let others deal with decisions”; “To continue to be oneself without focusing on disease and decision-making”.

**Conclusions:**

The findings underscore the need for flexible, person-centered approaches in SDM, tailored to the fluctuating health literacy and changing preferences of patients in palliative care settings. Our study contributes to the understanding of SDM in palliative care by highlighting how patients navigate the balance between autonomy and reliance on HCPs. Future research should explore how healthcare systems, including HCPs’ roles in the system, can adapt to the patients’ dynamic needs, to ensuring that SDM will remain a supportive and empowering process for patients at all stages of their disease.

**Supplementary Information:**

The online version contains supplementary material available at 10.1186/s12904-024-01434-2.

## Background

Globally, more than 50 million individuals are in need of palliative care annually, and this number will continue to rise in light of the aging population and advancements in medical care and treatment possibilities [1]. Patients in need of palliative care have an increased risk of critical incidents, hospitalizations, and deaths due to their complex illnesses [2]. This indicates a need for life-prolonging or symptom-relieving treatment clarifications, such as cardiopulmonary resuscitation (CPR). As such, shared decision-making (SDM), with distinct patient involvement in making decisions concerning their treatment and end-of-life wishes, is a crucial issue to emphasize. SDM entails a collaborative process between healthcare professionals (HCPs) and patients to make decisions based on the best available evidence and patient's preferences and values [3]. The significance of patient involvement in SDM is widely acknowledged, and many patients want to participate in decisions about their care and treatment, albeit at various levels [3]. SDM promotes a democratic perspective for patients in the palliative phase, as it fosters patient autonomy and freedom of choice [2].

Palliative care is a specialized medical approach that aims to improve a patient’s quality of life when living with a serious illness, regardless of the underlying diagnosis. Its main goal is to relieve physical, emotional, and psychological symptoms [4]. Traditionally, palliative care is considered essential during the final months and weeks of a patient’s life. However, this has changed with the recognition that the palliative phase extends beyond end-of-life care and that proactive decision-making significantly influences the patient’s overall disease trajectory [5]. To ensure patient involvement in decisions about their health and wishes for the future, adequate information about treatment options and symptom relief is deemed an essential prerequisite for patients in need of palliative care [6]. Dhollander et al. [7] found that patients experienced benefits from discussions with nurses concerning their preferences for the future. Nevertheless, their preferences regarding how they wished to be involved in the decision-making processes were not explored, nor was the significance of reflecting on their wishes clarified. Dhollander et al. [7] also emphasized the importance of *advance care planning* (ACP), which is the creation of a plan for treatment intensity in the event of a worsening health condition based on the patient’s values, goals, and preferences [5, 8]. Using such approach, Dhollander et al. [7] underline the importance of an early integration of palliative care in the patient’s treatment from a home care perspective.

 In Norway, facilitating palliative care services in the home is an overarching goal for healthcare policymakers and providers [9, 10]. The responsibility for health and care services is divided between state and municipal authorities, and the organizing of palliative home care services (HCS) varies across municipalities. Given the involvement of HCPs in SDM for home-dwelling patients in a palliative phase in Norway, there are some of importance to mention. Among them, general practitioners (GPs) play a crucial role in palliative HCS, as they are responsible for the medical treatment and follow-up of the patients. Additionally, nurses in HCS and cancer coordinators in the municipality are seen as key professionals in the follow-up care of patients during the palliative phase, often serving as a bridge between the patient and other healthcare services.

Previous studies have documented the SDM experiences of both patients and HCPs during discussions clarifying treatment in hospitals [11–13], nursing homes [14–16], and hospices [17]. However, further information is needed regarding SDM initiators and approaches in the context of home-based palliative care, particularly from the patients’ perspective [18]. The literature provides limited information about the proactive SDM experiences of palliative care patients receiving HCS. Therefore, the aim of this study is to investigate how home-dwelling patients in the palliative phase experience their involvement in SDM, and how they prefer to be engaged. By understanding the elements influencing patients' desire for active participation in SDM, we can improve the overall experience and outcomes of home-based palliative care, which is important in healthcare service planning.

## Methods

### Research design

A hermeneutic approach was selected as a suitable methodology for this study to emphasize the significance of the interpretation and development of a new understanding of the SDM phenomenon [19]. Individual in-depth interviews were conducted to collect data, in a more personal and exploratory manner, on the respondents’ thoughts, experiences, and perspectives on SDM, in order to thoroughly understand them [20].

### Participants, setting, and data collection

A purposive sampling strategy was used to recruit participants who met the following inclusion criteria: (1) diagnosed with a life-limiting illness in the palliative phase, (2) over 18 years old and capable of providing informed consent, and (3) willing to share their experiences related to SDM. The head of health and care services in each of six Norwegian municipalities was contacted to request support for inviting patients to participate in the study. The first author provided the heads of the health and care services both oral and written information on the study. Due to their limited response, cancer coordinators in the first author’s network were asked to assist in the patient recruitment. Furthermore, nurses in the HCS associated with local and regional palliative networks were contacted with the same request. These measures resulted in the recruitment of 13 eligible patients in the period from November 2022 to May 2023, as shown in Table 1. Three more patients agreed to participate in the study, but they decided to withdraw due to fatigue and their discomfort with the audio recording of the interview. Two more patients were identified as potential participants but were not approached due to the acute deterioration of their health, which led to their death.

**Table 1:** Study participants – socio-demographic data

	**(*N* = 13)**
**Sex**	
Female	8
Male	5
**Age**	
< 60 years	3
61–70 years	0
71–80 years	5
81–90 years	3
91–100 years	2
**Housing**	
Living with spouse	5
Living alone	8
Urban	8
Rural	5
**Community health care support**^**a**^	
GP and HCS	13
CC	12
PCT	6
**Diagnosis**	
Malignant disease	12
Non-malignant disease	1

^a^*GP* General Practitioner, *HCS* Home Care Services, *CC* Cancer Coordinator, *PCT* Palliative Care Team

Individual in-depth interviews were utilized as a data collection tool [20]. These interviews were conducted by the first author and were held in a quiet and comfortable setting for the patient, preferably in their own home. This allowed them to express their thoughts and experiences openly. The interviews varied in duration, ranging from 38 to 73 min.

The interview guide was developed for this study based on relevant literature and then, refined through pilot testing. The interview guide explored various aspects of SDM, including patient involvement, information exchange, and SDM preferences. Examples of issues guiding in the interviews were; *description of what the patient understand by the notion of being engaged in SDM regarding treatment and care; description of a situation where the patient experience being involved or not involved in such decision.* Probing and clarifying questions were used to elicit rich and detailed responses from the participants. Each interview was audio-recorded, and notes were taken to capture nonverbal cues and observations. The interviews were transcribed verbatim while ensuring the anonymization of the participants using pseudonyms.

### Data interpretation

Data were interpreted using a hermeneutic approach, guided by the method of Fleming et al. [21]. This method is based on Gadamer’s hermeneutical philosophy, in which the goal is to uncover a deeper meaning and develop a new understanding [19]. We created a dialogue with our pre-understanding and the data within the hermeneutic circle [19, 22], to contribute to a more nuanced understanding of SDM. To gain familiarity with the data, the transcribed texts from the interviews were read multiple times. The first and last authors read all the materials separately and noted the initial impressions, questions, and key concepts that emerged from the data. Next, the materials were analyzed sentence by sentence to unveil the nuanced meaning of each expression. This interpretive approach paved the way for the comprehension of the overarching message of the text, which required a back-and-forth process between the whole and its parts, and then back to the whole again, according to the hermeneutic rule of movement [19]. To ensure rigor and to enhance the credibility of the findings, all three authors actively engaged in reflective discussions throughout this process. All the initial findings underwent rigorous scrutiny to uncover empirical evidence that could challenge or invalidate any preliminary interpretations. By being open to our pre-understanding, we engaged in a more meaningful dialogue on the subject matter and arrived at a richer and more nuanced understanding of it. This iterative approach, grounded in hermeneutic methodology [19], ultimately led to our identification and articulation of the study’s results.

### Pre-understanding

Gadamer’s [19] perspective on pre-understanding emphasizes the role of prior knowledge and personal experiences as integral components of the research process. By acknowledging and critically examining these aspects, researchers can attain a deeper level of comprehension and interpretation. We, as researchers, were not detached from and impartial to the subject matter but were deeply committed to it. As such, in line with the principles of transparency [23] and trustworthiness [24], we had to make our pre-understanding evident in this paper. Our research team consisted of a professor with expertise in palliative care, an associate professor with formal and clinical qualifications in the same domain, and a PhD candidate with substantial clinical experience related to the patient group and the study context. According to our pre-understanding, we presumed that patients in the palliative phase receiving HCS are rarely invited to participate in SDM. However, during the interpretation phase of this study, the participants’ perspectives challenged these initial assumptions and led to a new understanding and deeper meaning of the subject.

### User involvement

To ensure user involvement in this study, we established a steering group and a reference group, inspired by the PAICPAIR framework (Patient and Informal Caregiver Participation in Research) tailored for research in a palliative context with vulnerable patients [25]. The steering group consisted of a patient in the palliative phase, and two researchers, which continuously evaluated the research process based on the input from the reference group. The reference group, consisted of a patient in the palliative phase, a nurse in HCS, a cancer coordinator, and a GP, which provided valuable inputs that shaped the research process positively by allowing us to obtain rich data. For example, the group provided valuable feedback on the information letter to the participants, which led to changes in the language to make the content more easily understandable to the recipients. Additionally, they added some questions to the guide about the patients' experiences in collaborating with HCPs.

## Results

**Fig. 1:**
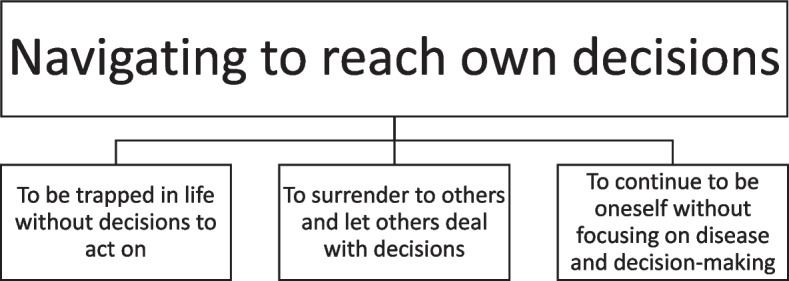
Results divided into categories

We identified the following overarching theme of the interview responses “Navigating to reach own decisions,” which emphasizes how home-dwelling patients in the palliative phase understand their involvement in SDM. Then, the participants’ responses as to how they desired to take ownership of crucial decisions in their life were categorized into three subthemes, as shown in Fig. 1: “To be trapped in life without decisions to act on,” “To surrender to others and let others deal with decisions,” and “To continue to be oneself without focusing on disease and decision-making.”

### To be trapped in life without decisions to act on

Several patients described experiences of being trapped in life without having control of decisions related to their ongoing treatment and potential treatment limitations. Living with a serious illness and knowing of their impending death were difficult to cope with, especially because they could not influence it to any extent. The experience of living with such uncertainty led to feelings of resignation and generated several existential questions:

*Well, it’s the uncertainty that’s difficult. Not knowing what’s ahead, you know. (...) I’ve stopped planning, and then someone says you could live for many years, but I don’t know. (…) Just being here at the day center, it’s a challenge because there are patients here who are worse off than me, and they’re dying, you know, yes, and here I am, what am I doing here?* (Woman, 56)

The feeling of losing control over one’s life led to an experience of merely existing. Several informants explained this as the loss of opportunity to influence one’s future. This challenged their need to own their decisions. While some described resignation, others explained how they wished to regain control through decisions such as opting for assisted dying. At the same time, the statements highlight the suffering the patients experienced and how this suffering led to resignation despite their desire for improvement:

*I don’t have a life. I just exist. And it’s a matter of how long I bother or have the energy. (…) No, if I don’t get better from this [treatment], I’m almost inclined to apply for when they end your life. Assisted dying. Because there’s no life, like this. If this doesn’t work, if it goes on and on and on like this, I don’t want to live anymore, and I mean that.* (Woman, 73)

Several patients described how feeling trapped in life made them want to have control. However, having control did not necessarily mean having control over the future. Some participants described how they saw death as a better alternative than life and that they had resigned themselves to the fact that their life was nearing its end. One patient considered death a relief when one felt trapped in life and how crucial it was for the patient to have a voice concerning this decision:

*I’ve come so far in life that I’ve done what’s expected of me. Now I can’t do anything more for my children and grandchildren. They must manage on their own, and I need to be allowed to make decisions for myself. If I’m allowed to decide, then I say please let me die now. (…) This isn’t a life. To live just for the sake of living, no one should have to do that.* (Woman, 81)

Several participants described the choices they had made regarding their treatment limitations, such as the “Do not attempt resuscitation” (DNAR) choice. Even though the patients wanted to have control over the important decisions in their life, this was not a reality for most of them, as they rarely discussed their wishes with HCPs. Several patients described it as unnatural to be asked about their treatment preferences, such as DNAR, by the GP with whom they had only sporadic contact, since they were primarily followed up in specialist healthcare service facilities. Several patients had not thought about the issue with DNAR but took it for granted that CPR was not applicable in their situation. At the same time, some patients said they did not want to be asked about resuscitation as they did not want to consider it. Furthermore, the patients described their conversations with HCPs as fragmented, and most of the patients apparently experienced the practical role that HCS played but did not know whom to approach about their further treatment and follow-up. An old man described how different HCPs had different roles and how he did not find it natural to talk about CPR nor to lay the responsibility on HCS or the GP for providing him such information:

*In today’s situation, I would just let it [the heart] stop. Yes. I have a clear opinion concerning this. I’ve had a good life, and I’m really old. Yes. (...) well, it’s the practical stuff that they [HCS] help me with. That’s what it is. But I wonder if there’s any point [in carrying] out . . . that heart examination, [in checking out] that tight valve. I have to ask [the GP] about that.* (Man, 94)

### To surrender to others and let others deal with decisions

The patients described how their ownership of decisions could be expressed in various ways. Several decisions need to be made concerning their treatment throughout the palliative care trajectory, and it was important for many of them to rely on HCPs’ knowledge when important decisions related to their further treatment had to be made. The data showed that the patients had faith in the doctor in charge of their treatment and entrusted to the medical staff the making of crucial decisions for them. Simultaneously, several patients experienced having had to make burdensome choices they felt they could not make, and if they had to decide, they expressed a need for proper information:

*I was asked if I wanted radiation or if I wanted chemotherapy. And for some reason, I panicked about that radiation, I definitely didn't want that. So, I chose chemotherapy. But if I had known what I know today, with the neuropathy and everything, of course, I would have chosen radiation. But that’s my own fault.* (Woman, 73)

Some patients regarded entrusting decisions to others as relying on others to give them enough information so that they could make the decision themselves, whereas other patients regarded it as giving HCPs the authority to make decisions for them. It was unclear whether the patients wanted to make the decisions themselves or wanted the HCPs to decide. Also, the level of influence of HCPs in making these choices varied significantly. The patients also expressed the powerlessness and vulnerability they felt when placing their lives in the hands of others:

*I could have taken both chemotherapy and radiation if I wanted to, they wouldn’t have denied me that. (...) I’m actually completely helpless. I’m totally dependent on those who know something about this and can do something.* (Man, 81)

As the patients’ illness and treatments made them weaker, they relied more on the HCPs, with their expertise, to make the decisions concerning their possible treatments. Some patients described how they had to live with the consequences of the choices made, and some patients felt that their treatment refusal was not taken seriously. One patient described how she gave in because she could not handle the doctor’s insistence:

*They thought I should try treatment first before I could say anything. Yes, that might be the case, but my standpoint is not to accept any treatment. They had problems understanding that. “It’s important to try,” the doctor said. He kept going on like that. Eventually, I said yes. (...) so I was there at the hospital alone, waiting for something to happen, but no, I was told that I was too sick to undergo those treatments.* (Woman, 75)

### To continue to be oneself without focusing on disease and decision-making

It was important for the patients to continue being themselves, regardless of their illness and impending death. This was expressed in how the patients owned their choices and how this ownership was significant to them. They described the importance to them of being acknowledged and seen by HCPs, as it gave them a sense of being more important than their illness. Being taken seriously and spoken to in a language they understood contributed to the patients’ ability to be themselves.

*I think the most important thing is being taken seriously. That it’s not just a doctor sitting there brushing it off when the illness is that serious. (…) That they have time, and understanding, that they respect the communication, that’s very important. That I understand what’s being said, that it’s not just like columns and ... yes, jargon.* (Woman, 56)

Most patients described having frequent meetings with HCPs during their illness; however, they also described how they rarely felt they were given the opportunity to talk about their feelings and thoughts about the future. Many of the patients experienced long and tiring illness trajectories and expressed how they regretted that they did not take the initiative to be more open about their illness experiences and thoughts related to decisions about their future. Some of the patients indicated a desire to be asked by HCPs how they were doing, while others placed the responsibility on themselves:

*No one has asked, “How are you doing?” But then again, I haven’t pushed for it either. (…) I’m really happy when someone asks, then I can decide if we can talk some other time or if it’s okay to talk now.* (Man, 58)

Several of the patients highlighted individual HCPs, such as a nurse in the HCS or a cancer coordinator, as important supporters and conversation partners regarding decisions and everyday-life issues. The results showed that often, it was simple and practical aspects that contributed to making the patients feel seen and heard, such as offering to move a bed to a more convenient location or volunteering to make a phone call to inquire about test results without the patient having to ask for it. At the same time, several patients emphasized the importance to them of having deeper conversations with persons they had come to trust. However, a prerequisite for these conversations was for both parties to talk as humans and not within a formal patient–HCP relationship:

*I usually say that the best medicine for a person is another person. And I think there’s a lot to that. It’s the sense of security you get. So, how healthcare professionals convey themselves through body language is very important.* (Woman, 79)

## Discussion

The aim of this study was to investigate how home-dwelling patients in the palliative phase experience SDM, and how they prefer to be engaged in it. Our findings revealed that the patients had a common overarching goal regarding their SDM experiences and preferences: “navigating to reach their own decisions.” Their goal was not merely to own their choices but to navigate major decisions in life. This might be understood as more than merely choosing between prolonging life or alleviating symptoms but deciding on matters of life and death amid the fear of maneuvering in an unknown landscape. Within this terrain, it is crucial to highlight the content of White Paper no. 24; *Palliative care and treatment – Some day we will all die. But on all other days, we will not* [26], which emphasizes that everyone should be able to choose where to stay when nearing end-of-life and express their preferences and life goals. This viewpoint has been characterized by an increasing emphasis on the user perspective, which promotes an equitable, democratic collaboration well suited to human rights [27]. This perspective strengthens the role of patients in SDM but also challenges the demands placed on them. In light of our findings, patients experience having to make a number of choices they could not make, which compels them to live in uncertainty.

Several patients said they felt trapped in life, which led to resignation and a sense of losing control. This feeling of losing control over one’s life could lead to an unsettling feeling that one is merely existing, which complicates one’s navigation of the already challenging palliative phase of making and owning one’s decisions. This aligns with a systematic review [28] that highlighted that patients’ maintenance of a sense of control is associated with patient empowerment. In this context, the patients’ goal was not to have control over their illness but to assert their self-identity and to feel respected in relationships with HCPs. Additionally, patients manifested control by delegating specific practical tasks to their family members or to HCPs so that they could preserve their energy for matters that were important to them [28]. Likewise, our findings showed that some patients manifested empowerment by consciously assigning decision-making power to HCPs. Power delegation can still give individuals a sense of ownership over their decisions, as they have confidence in the competence of the HCPs. In addition, leaving decision-making processes to others might be seen as a relief for some patients. Such handing over is a way of expressing trust in the ability of those in charge to take responsibility for making the decisions [29]. On the contrary, other studies emphasized the importance of supporting the patient’s autonomy by preventing them from leaving decisions to HCPs [30, 31]. For instance, this dichotomy is seen in situations in which clinicians consciously attempt to influence a preference for treatment versus situations in which clinicians affirm the patient’s autonomy. Kuosmanen et al. [18] supported these findings, indicating that patients receiving palliative care desire greater participation in decision-making beyond merely on medical options. In addition, Sandsdalen et al. [3] emphasized how patients’ participation in decision-making enables them to retain control over their own lives and over their care. This might be understood in the traditional context of SDM as centered on the patient–physician relationship [32] and confined to medical choices [33]. This traditional view is also applied in the palliative care context [30, 34]. However, based on the patient narratives about what these decision-making processes entail, there appears to be a need for a broader understanding of who should be involved in SDM in the context of home-based palliative care. For example, it was found that the sense of control is influenced by decisions regarding treatments that are consistent with one’s personal values and goals [35]. In this study, the patients revealed that they often took a clear stance based on their beliefs and ownership of their choices regarding treatment limitations. Despite their desire to have control over their life decisions, this was not the reality for most of them. They were seldom offered the opportunity to discuss their future perspectives with responsible HCPs, and they related this to their experiences of the somewhat unclear roles of HCPs in SDM. This might negatively influence the patients’ navigation in this landscape, as they experienced difficulties in knowing whom to approach. It is therefore puzzling that our findings suggest patient involvement in SDM regarding, for example, treatment clarification. As shown, our study uncovered a gap between patients’ desire to exert control over their treatment decisions and the reality of the somewhat unclear roles of HCPs in SDM. These findings are similar to those of Bélanger et al. [36], who suggested that HCPs face a marked challenge in talking about decisions involving the acknowledgment of an impending death. The conversations about symptom relief—an often seemingly straightforward topic—that HCPs initiate are directly related to improving the patient’s immediate well-being. Comparable perspectives are found in the study of Rodriguez et al. [37], which stated that HCPs often seem to avoid conversations about the future and death. The complex existential challenges that patients in the palliative phase face contradict the system that HCPs work under, which is characterized by plans, guidelines, and a pathway-oriented mindset [37]. Our findings indicate that deeper, trust-based conversations are important, preferably in a more informal, human-to-human interaction beyond the typical patient–HCP dynamic. This appears to be a challenge in a context driven by a procedural mindset and standardized frameworks. Standards and systems may function like maps that ease navigation. However, in this context, rigid systems lose sight of the patients’ perspectives and choices in navigating in their tempo and regarding their preferences. Our findings underscore the need for HCPs to initiate conversations about patients’ preferences and values. In this context, ACP may be a relevant method [6].

ACP is a proactive person-centered approach that involves meetings between the patient and the HCP on the patient's values regarding their future treatment and care [6]. Although ACP is referred to as a “method,” it can also be used as a process for reflecting on the meaning and consequences of a serious illness. Therefore, in this context, the concept of a person-centered SDM framework is relevant to discuss further. This perspective emphasizes that SDM should be viewed as a comprehensive collaborative process in clinical care rather than being limited to treatment options [38]. This perspective requires clinicians to deeply engage with patients’ experiences of their illness and their involvement in treatment. SDM also incorporates the knowledge, experiences, and values of all the participants so that the decision-making could be shared. Knowing the patient as a person and providing an autonomy‐supportive context for care are crucial [38]. Related to the goal of palliative care and in line with our findings, decisions are more than merely treatment choices, as they extend to fundamental concerns about life and death. Pask et al. [39] highlighted the importance in palliative care not only of addressing traditional physical, psychological, social, and spiritual aspects but also recognizing and managing preexisting and evolving complexities, unseen factors, systemic influences, and societal impacts. This illustrates the complexity of palliative care, in which SDM takes on a distinct character due to its holistic approach, which is considered a fundamental aspect of palliative care [18].

Person-centered SDM involves mutual respect between the HCP and the patient, in which the HCP knows the patient so well that they can answer the patient's question, “What would you do if you were me?” [38]. This understanding empowers patients to exercise their autonomy [18], which, in turn, allows them to assert control over the decision-making process [40]. We recognize that for patients to have control over life-altering decisions, such as treatment limitations, they must be given the opportunity to engage in discussions with a responsible HCP [41]. These discussions should be conducted in a way that highlights the patients’ dignity and preferences [42], and if possible, include a next-of-kin to strengthen the patient’s perspectives in communication with HCP [43], which, in turn, support the patient’s navigation during the palliative phase. Many patients in palliative care face challenges in communicating with HCPs to be able to understand and use the information provided [44], often due to their limited health literacy [44, 45]. *Health literacy* refers to a person’s ability to understand, process, and use health information effectively [45]. Our findings indicate the importance to patients of experiencing ownership of decisions, being taken seriously as a patient, and being spoken to in a language they understand. Limited health literacy can be a contributing factor to these needs, as it can hinder patients’ autonomy and effective participation in SDM [46]. In this study, some patients expressed that they felt ill-equipped to make complex healthcare decisions, which underscores the critical role of health literacy in SDM. In palliative care, where decisions often involve intricate medical information and profound personal values [39], the lack of prerequisites for making informed choices can be particularly impactful. Based on our findings, this lack challenges healthcare systems to enhance health literacy to ensure that patients are supported in navigating and understanding their healthcare options and in recognizing when to delegate decision-making. Our findings indicate that entrusting healthcare decisions to HCPs can lead to a sense of ownership over choices. This seems to contradict person-centered care principles but might be in harmony with these principles in certain contexts. Patients often place immense trust in the competence of their HCPs, and this trust can manifest as a sense of ownership of the decisions made by these professionals [47]. This finding aligns with recent shifts in SDM models wherein the decision-making process is viewed as a collaborative journey rather than a moment of independent choice [38].

Improving health literacy is essential, as it directly impacts a person’s capacity to actively participate in their healthcare [45]. Nonetheless, Schultz and Nakamoto [48] raised concerns about relying solely on a patient’s level of health literacy indicative of the patient’s level of empowerment. They argued that a high level of health literacy does not necessarily equate to a high level of empowerment. Even if patients know the pros and cons of various treatment and care options, this knowledge may not be sustainable unless the patients’ level of empowerment allows them to express it. In any case, Tonelli and Sullivan [38] highlighted the importance of the nature and extent of the information given to each patient and how it should be individually tailored by the HCP. This underscores the need for flexible, person-centered approaches in SDM tailored to patients’ fluctuating health literacy and changing preferences in palliative care settings. Such an approach helps patients navigate the balance between autonomy and reliance on HCPs. Yet, Pilnick [49] critiqued the implementation of patient-centered care in healthcare, suggesting that while patient-centered care aims to combat medical paternalism and enhance patient autonomy, it often leads to patient abandonment in decision-making. Reflecting on our findings, this argument seems relevant. However, it is worth questioning whether the outcome would be different if patients were approached with patient-centered care combined with SDM in a way that truly shares decision-making [41]. Our study indicates that this was not always the patients’ experience; they highlighted the importance of being allowed to not always engage with HCPs on all decisions, as this can also give them the feeling of owning their decisions. At the same time, our findings show that there is a difference between being asked to make medical decisions and being asked about future preferences. This raises questions about whether we are placing high demands on patients’ ability to navigate the terrain when they are vulnerable during a demanding period of illness, especially when they themselves report being responsible for initiating conversations regarding such decisions. This aligns with Pilnick’s [49] argument on the need to differentiate between medical expertise and authority, and suggestion to recenter medical expertise in healthcare.

We highlight the need for a nuanced understanding of patient autonomy and decision-making. It calls for a balance between respecting patient autonomy and providing expert guidance, especially in complex and emotionally charged settings such as in palliative care. Ultimately, this debate opens avenues for redefining roles and responsibilities in home-based palliative care and for advocating a more collaborative and empathetic approach that values both patient preferences and professional expertise. In this manner, HCPs can contribute to increasing patients’ sense of ownership of their decisions and ability to navigate their own decisions.

### Limitations

We aimed to recruit patients in the palliative phase, regardless of their diagnosis. The final sample included only one patient with a noncancer diagnosis, possibly because cancer coordinators assisted in the recruitment of participants. Although this could have limited this study, the primary focus of this study was patients in the palliative phase, with their specific diagnosis merely secondary. Moreover, the interview transcripts were not returned to the participants for their review and comments, and the study results were not presented to them for their feedback on the alignment of the interpretations of the results with their personal stories. However, we had decided not to present the results to them so as not to further burden them, given their health condition. Additionally, several patients passed away before the preliminary findings could be compiled. Therefore, we presented the findings to the reference group, who gave us feedback. As in all qualitative research, our findings are inherently tied to the text and limited by the contexts of the participants and of the study setting, which, in this study, is Norway. However, to increase the transferability of the results, we provided rich descriptions of the patients’ experiences. While patients were the primary concern of this study, it's important to recognize that an increased attention given to caregivers, particularly in light of the holistic principles of palliative care, would have given richer data. Nevertheless, by solely refining the patient perspective in this study, it provides a unique depth to the data material.

## Conclusion and future implications

To our knowledge, this study is the first to specifically explore how home-dwelling patients in the palliative phase who were receiving assistance from HCS became involved in SDM and how they preferred to be engaged therein. The results were grouped into three subthemes on the patients’ experiences of the importance of owning their choices. They expressed a desire to maintain control of their treatment despite feelings of resignation, preferred entrusting decisions to HCPs, and emphasized the importance of being recognized as whole persons. In summary, this study contributes to an understanding of SDM in palliative care that highlights how patients navigate the balance between autonomy and reliance on HCPs. It underscores the need for flexible, person-centered SDM approaches tailored to the fluctuating health literacy and changing preferences of patients in palliative care settings.

Future research should explore how healthcare systems, including HCPs’ roles in the system, can adapt to patients’ dynamic needs to ensure that SDM will remain a supportive and empowering process for patients at all stages of their disease.

### Supplementary Information

**Supplementary Material 1.**

## Data Availability

No datasets were generated or analysed during the current study.
