# Supplementary material for: Patients’ experiences with shared decision-making in home-based palliative care – navigation through major life decisions
Source: BMC Palliat Care. 2024 Apr 17;23:101. doi: 10.1186/s12904-024-01434-2 (PMC11022472; doi:10.1186/s12904-024-01434-2)
Supplement: Supplementary file 1 — Supplementary Material 1. [file 12904_2024_1434_MOESM1_ESM.docx]

**Semi-structured modifiable interview guide:
Patient (sub-study 1)**

**Opening questions:**

- Can you tell me a little about your situation?
- I would like to hear what you associate with the concept of treatment clarification?
- How do you interpret a decision-making process?
- How do you interpret the involvement of a decision-making processes related to your further treatment?
  - When healthcare professionals talk about your treatment now and in the future, to what extent do you experience that they ask for your opinion?
- Can you tell me about a situation where you felt involved in decisions about your treatment?
  - What was this experience about?
  - Can you elaborate on your experiences/perspectives?
- Can you tell me about a situation where you felt *not* being involved in decisions about your treatment?
  - What was this experience about?
  - Can you elaborate on your experiences/perspectives?

**Your relationship to homecare nursing / view on the role of homecare nursing**

- To what extent do you experience that homecare nursing is involved in your situation?
  - How frequently are you in contact with the homecare nursing?
  - In what way do you experience that they are interested in your situation?
  - In what way do you experience that they are updated regarding your situation?
- Do you feel that the homecare nursing takes responsibility for your follow-up and your overall situation?
  - To what extend have healthcare personnel taken the initiative to discuss the future with you?
  - If yes: How did you experience this?
  - Can you describe how this was done?
- What is your experience concerning the collaboration of homecare nursing staff and your GP when it comes to your situation?
- Do they help you with what is important to you?
  - If no: What are your thoughts around the lack of cooperation?
  - If yes: How do you experience this cooperation?
  - What are your wishes in terms of the homecare nursing and GP cooperation?
  - How does the cooperation between homecare nursing and the GP affect what is important in your situation?
- Do you have contact with a cancer coordinator?
  - If yes: what is her/his role in relation to your situation?
  - How do you experience that the cancer coordinator makes plans together with you?
    - Can you describe how this was done?
  - How do the cancer coordinator, homecare nursing, and your GP work together regarding your situation?
    - How do you experience this collaboration?
    - How would you like this collaboration to be?

**The relationship with your GP / the GP's role**

- What experience do you have concerning the contact with your GP?
  - How long have you had your GP, and how often do you see him/her?
- To what extent do you want your GP to ask you about your wishes for the future?
  - To what extent do you want your GP to involve you in treatment alternatives?
- Have you experienced that your GP has initiated a conversation related to clarifications about further treatments?
  - If no: how would you like your GP to do that? Have you yourself initiated such a conversation?
  - If yes: how did you experience this conversation?
  - Can you describe how this was done?
- Has your GP ever asked you if you want to be hospitalized in case of worsening of your health condition?
  - If yes, in what context was this brought up?
  - To what extent do you want your GP to involve you in decisions about hospital admissions?
- Has your GP ever asked you if you want to be resuscitated if your heart stops?
  - If yes, can you tell more about that conversation?
  - To what extent do you want your GP to involve you in decisions about resuscitation?
- Now that you live at home and your GP has the medical responsibility for you, to what extent do you feel safe?
  - Can you elaborate on this?
  - Related to the relationship between you and your GP, have you ever felt insecure about the medical responsibility?
    - Can you elaborate on this?
- Have you ever wished that your GP, in cooperation with the homecare nursing or the cancer coordinator, would make decisions related to your treatment on your behalf?
  - Can you elaborate on this?
- Can you say something about what feels meaningful to you now that you have a disease that you cannot recover from?
- What will be important to you when life is nearing its end?
  - In what way do you want others to be involved in your thoughts about the end of life?

**Conclusion/Summary**

- We are now approaching the end of the conversation. What do you think is the most important thing we have talked about today when it comes to decision-making processes?
- Is there anything we have not talked about, that you think is important to highlight about decision-making processes and treatment clarifications around your disease situation?
